# A College Knowledge Program for Latino Immigrant Families: Examining Parental Academic Involvement and Adolescents’ Academic Goals

**DOI:** 10.3390/socsci8100275

**Published:** 2019-09-29

**Authors:** Griselda Martinez, Gabriela Chavira

**Affiliations:** 1 Department of Human Development and Family Studies, The Pennsylvania State University, Philadelphia, PA 16802, USA; 2 Department of Psychology, California State University, Northridge, CA 91325, USA;

**Keywords:** Latina/o, immigrant families, adolescents, college knowledge, parental involvement, academic goals

## Abstract

The current study examined how parents’ and adolescents’ reports of parental involvement were associated with adolescents’ academic goals before and after participating in a college knowledge program. Twelve parent-adolescent dyads (M_age_ = 13.58) participated in the program. Thematic analysis was used to analyze these data and create themes that emerged based on patterns in parents’ and adolescents’ semi-structured interviews. Findings suggested that while parents’ reports of their involvement remained relatively the same (high involvement), half of the adolescents indicated increases in their academic goals and perceived parental involvement after participating in the program. This study highlights the role of a college knowledge program on parents’ and adolescents’ changes in perceived parental involvement and academic goals. The study findings identify an avenue to help families access additional capital that can help their children pursue their academic goals.

## 1. Introduction

In 2012, 49% of Latino high school graduates enrolled in college, and for the first time surpassed the college enrollment of Whites (Lopez and Fry 2013). Since then, the number of Latino graduates enrolling in college has continued rising (Gramlich 2017), with a 67% immediate college enrollment rate in 2017 (McFarland et al. 2019). Despite these gains, Latino students have lower educational attainment compared to other groups and are over-represented in two-year colleges (Krogstad 2016; National Science Foundation, National Center for Science and Engineering Statistics 2019). Providing Latino families and youth an opportunity to learn the tools and information about the process and requirements needed to pursue a post-secondary education is important to guide students towards college admission (Gandara and Contreras 2009). However, prior work on college information programs has either focused on adolescents (Mendiola et al. 2010) or parents (Auerbach 2004; Chrispeels and Rivero 2001; Parent Institute for Quality Education), but rarely have programs included both parents and their children. Parental involvement plays an important role in Latino youth’s academic outcomes (Ceballo et al. 2014), despite challenges that parents may encounter due to cultural mismatches between home and school expectations (see Zarate 2007). According to a meta-analysis by Fan and Chen (2001), ‘parental involvement’ has been widely defined as parent-child communication, home supervision, educational goals for children, and school contact and participation, resulting in inconsistent findings on adolescents’ academic achievement. Yet research on Latino immigrant parents has found that families consistently hold high academic goals for their children (Ceja 2004), highlighting the existing capital and strengths among immigrant Latino families. Immigrant parents, however, may lack general information on the US education system and post-secondary options (Hernandez et al. 2007). Using Social Capital (Coleman 1988) and the Community Cultural Wealth Model (Yosso 2005) as guiding perspectives, we hope to reveal the mechanisms of changes in parental involvement that contribute to students’ academic goals via a college knowledge program.

### 1.1. Theoretical Framework

Capital theories address how an accumulation of cultural knowledge, skills, and abilities are possessed by privileged groups in society and how the result of knowledge obtained through family or formal schooling is often limited for working-class families who may experience minimal access to social mobility (Bourdieu and Passeron 1977). Within the family context, Coleman (1988) suggested three components of capital that include: financial capital, family income, physical resources, and space for children to learn and the availability of monetary funds; human capital, parents’ educational background; and social capital, relationships, and communication that exists between parents or family relatives and the children. Coleman argued that social capital is a strong asset for families and children, and without social capital, even if there is a low or high source of human capital, human capital becomes irrelevant to a child’s educational growth. Social capital also extends beyond the family and includes the social relationships that exist among parents and their relationships with institutions present in their communities (Coleman 1988). Salas (2016) argued that college knowledge is a form of capital that many Latino parents do not have access to which can drastically limit a student’s chances of attending college. Studies using traditional perspectives of social capital have found that compared to White students, immigrant Asian and Latino students as well as Black students possess less social capital, including parental involvement (Kao and Rutherford 2007). Similarly, studies on Latino populations have examined parental involvement as a form of social capital by examining mechanisms through which parental involvement may contribute to Latino adolescents’ academic outcomes (McNeal 1999; Kuperminc et al. 2008). For example, Kuperminc et al. (2008) found that for Latino high school students, perceived school belonging, and teacher expectations mediated associations of parental involvement with academic adjustment.

A traditional perspective of social capital may not be sufficient to understand the unique contributions that Latino families make to help their children succeed. For Latino communities, there are additional forms of capital that equip families with information and resources. The Community Cultural Wealth model (Yosso 2005) shifts away from a deficit view of communities of color and highlights family assets and sources of information that include social, aspirational, linguistic, familial, navigational, and resistant forms of capital. More specifically the current study focused on three of Yosso’s forms of CCW, ‘social capital’ which entails networks of people and relationships with sources present in their communities that provide information about how to navigate social institutions, ‘aspirational capital’ which represents the ability to maintain hope for future dreams and possibilities even in the face of challenges, and ‘navigational capital’ which refers to the ability to maneuver through social structures that may be challenging for individuals to traverse through such as university campuses. These cultural assets are imperative for families and play an important role in helping their children succeed (Chavira et al. 2016). These sources of capital work together and play a dynamic and important role in the multiple strengths that families possess as they attain and transmit sources of information and traverse through social structures such as the educational system with their children (Yosso 2005). The current study utilizes the CCW as a mechanism to better understand Latino families’ sources of information, including different forms of capital, and how these families seek information that will help them and their children as their children pursue higher education.

### 1.2. Accelerators to Paths of Success—The Role of Families

Adolescents’ families, teacher relationships, and opportunities to excel in school through bridging programs and knowledge on how to prepare for higher education contribute to students’ access and motivation towards higher education (Cooper et al. 2011). Previous studies examining the association between parent behaviors and their children’s academic experiences have found that parents play an important role in their children’s academic experiences by providing academic support, assistance with school, and placing a high value on their academic success (Fuligni 1997; Plunkett and Bámaca-Gómez 2003). Supportive relationships between parents and adolescents, especially monitoring from parents has also been found to serve as protective factors for students’ academic achievement (Prelow and Loukas 2003). Latino parents and their children both place high values on college education (Immerwahr 2003; Lopez 2009) thus suggesting the important role that parents serve in their children’s academic goals.

### 1.3. College Knowledge Programs

Latino families, especially immigrants, experience challenges in acquiring and understanding information about the necessary steps that children must experience before enrolling in a university. Low-income students often have limited accessibility regarding information about university enrollment (National Research Council 2006; Schneider et al. 2013), which requires a network beyond Latino families (Kim and Schneider 2005). Despite these challenges, extant studies confirm that Latino families place a strong value on education (Chavira et al. 2016) and seek information from their social relationships (Tornatzky et al. 2002). College programs have also shown to improve college awareness when providing information to families about college admission, college testing, and the requirements and procedures for financial assistance (Downs et al. 2008). Prior studies of Latino parent participation in a pre-college program in Southern California, revealed that parents attained new information about the steps necessary for their children to enter post-secondary institutions, recognized the need for their continuous involvement during the process, built college-relevant social networks with other parents, and the value of a four-year college for their families (Auerbach 2004).

College preparatory courses have also been implemented in schools to prepare students with skills and information that can help them prepare for college. This includes programs such as Advanced Via Individual Determination (AVID). Mendiola and colleagues (Mendiola et al. 2010) examined the long-term impacts of AVID among students who were enrolled in a university. Findings revealed various themes that emerged from students’ interviews including having experiences of a rigorous high school curriculum, teacher intervention, and assistance with college and financial aid applications. While these college preparatory programs can grant students opportunities to develop knowledge and acquire skills that will prepare them when getting ready to apply for college admission, the majority of these programs focus on the adolescents only, are very expensive to run, and are available in limited schools.

In contrast, programs focused on educating parents have become accessible for families in high schools. The Parent Institute for Quality Education (PIQE) is a nine-week parent engagement in education program focused on informing parents about the U.S. education system, how to interact with schools and teachers, and how to help their children at home succeed in school. Chrispeels and Rivero (2001) examined the impact of PIQE, and found changes in parents’ communication with their family and children’s teachers such that parents reported that they learned how to work with their children in school-related activities and as a result experienced better communication with their children, increased their basic support for their children, and developed awareness of how to build their children’s self-esteem. Furthermore, a study on moral support in a college access program revealed that Latino immigrant parents were involved in their children’s academic experiences through conversations and moral support stressing the value of hard work, study, and education (Auerbach 2006). Parents who participated in the college access program also engaged in concrete actions to help their children succeed, including parents and their children commuting to the best school, and parents clearing away possible distractions such as chores or the need for their child to work to help families financially. Therefore, prior research highlights the need to provide both parents and adolescents with college information about the pathways to higher education to create meaningful experiences for families and allow them to become more knowledgeable on how their children can succeed in school.

The current study examined the role of a college knowledge program aimed to increase parental involvement and college awareness in low-income immigrant Latino families of middle school and high school students. This study used the Community Cultural Wealth model (Yosso 2005) to better understand the access of college information and how a program at a community-based organization for Latino families assisted families in their acquisition of college knowledge. This study examined parents’ reports of parental academic involvement, adolescents perceived parental academic involvement and adolescents’ academic goals before and after having participated in the college knowledge program. The following research questions guided this study: (1) How do parents perceive their parental involvement at home and school and how are these behaviors associated with adolescents’ academic goals before and after participating in the program? (2) How do adolescents perceive their parents’ involvement and how are they associated with adolescents’ academic goals before and after participating in the program?

## 2. Method

### 2.1. Participants

Sixteen families participated in the six-session college knowledge program. For the current study, only parent-adolescent dyads that provided active consent and participated in both the pre (T1) and post (T2) data collections were included in this study, which included one family with two siblings (1 girl, 1 boy). A total of 11 Latino parents participated in the pre and post portion of the college knowledge program (10 mothers, 1 father). All of the parents were born in Mexico and had an average age of 40.18. For parent education, parents averaged a middle school education (M = 7.33 years), with five parents (45%) reporting a 6th-grade education level or less, four parents (36%) had some high school education, and two parents (18%) graduated from college or university. Seven of the mothers (64%) were unemployed, three parents (two mothers and one father, 27%) worked part-time (i.e., housekeeper, restaurant) and one mother’s employment status was missing. A total of 12 Latino adolescents (one family had two siblings) participated in the study including six (50%) boys and six (50%) girls, (M_age_ = 13.58). Of the adolescents, nine (75%) were U.S.-born and three (25%) were born in Mexico. At baseline, seven (58.3%) of the adolescents were in middle school and five (41.6%) were in high school.

### 2.2. The College Knowledge Program

The six-session college knowledge program was developed and delivered by the research team, consisting of the authors and a master’s level student. The goal of the program was to provide parents and their adolescents with parallel information, resources, and tools about pathways to college that is not traditionally given in college preparation workshops. Whereas most Latino families and students are guided towards California state universities, we provided in-depth information that is not typically given to low-income families, such as finding the right college/university for your child, including private universities, finding universities that meet 100% of financial need, and strategies when applying to schools, (e.g., reach vs. likely to be accepted). The parent sessions included: (1) the importance of parent involvement; (2) parent rights at school; (3) differences between colleges and universities; (4) preparing for college; (5) finding resources in the community and internet; (6) financial aid. The adolescent sessions included: (1) planning and surviving high school; (2) classes matter; (3) the importance of college and differences between colleges and universities; (4) preparing for college; (5) letters of recommendation and taking charge; and (6) financial aid. Each of the sessions were delivered at the community-based organization (CBO) and took approximately 1.5 h each (for a total of nine hours of instruction). The program sessions were conducted every three weeks. All parent sessions were delivered in Spanish and the adolescent sessions were delivered in English (with a translator for students who were Spanish-dominant).

The research team partnered with a CBO in the Los Angeles Area. This organization provides a wide range of social services and educational classes for parents and adolescents including tutoring services, computer classes, and English as a Second Language (ESL) classes. All recipients of the services offered at the CBO have household incomes at or below the federal poverty line and lived in the CBO’s service area.

### 2.3. Procedures

The research team attended multiple events at the CBO to meet clients and provide information about the college knowledge program. The CBO coordinator also assisted with participant recruitment by distributing fliers.

Parents and their children were invited to participate in a semi-structured interview that took place at the CBO during their open hours. A total of 16 parents and their adolescents participated in the pre-program semi-structured interview and baseline survey, which took 15–20 min for adolescents to complete and 30–45 min for parents to complete. The program took place from January through May 2016. Of the 16 parent-adolescent dyads who participated in the college knowledge program, only 11 participated in the post-program semi-structured interview. Therefore, this study examined the information relevant to these 11 families. All participants completed informed consent and assent forms. Each participant was interviewed in a separate room. Trained researchers conducted the semi-structured interviews in the participants’ preferred language (English or Spanish) and the interviews were audio-recorded. Pseudonyms were used to maintain the anonymity and confidentiality of the participants.

The 11 families varied in their attendance of the 6 college knowledge sessions, ranging from 2 to 6 sessions attended, with a mean of 4.0 sessions for adolescents and 4.3 sessions for parents. The program included information about the University of California (UC) and California State University (CSU) course requirements for high school students, better known of A-G subject requirements. The program also included information on how to prepare during high school (e.g., college entrance exams, Honor and Advanced Placement courses), financial aid, how to request letters of recommendation, responsibilities in college, differences between post-secondary institutions (e.g., private, CSU, UC, out-of-state), and admissions requirements. Parents were also presented with information about how to remain involved during adolescence, parents’ rights in public schools, how to locate and access resources online, and how to help prepare their youth for college (i.e., college knowledge).

### 2.4. Measures

Parental Involvement: Participants took part in semi-structured interviews before and after participating in the program. The semi-structured interview questions were adapted from the Ecocultural Family Interview (Weisner et al. 1997). The pre-program and post-program semi-structured interviews for parents and adolescents included questions related to parental involvement that was perceived to be contributing to the adolescents’ academic goals and success.

To fully capture all the different ways parents contributed to their child’s education, we broadly defined parents’ and adolescents’ reports of ‘parental involvement’ as any parental behavior (e.g., communication, home supervision, school contact, and participation) that the participant perceived as contributing to the academic success of the child (see Fan and Chen 2001). We separated parents’ reports of academic goals from parental involvement as academic goals are shown to be strong predictors of academic success (Fan and Chen 2001) and may not always be communicated to the child (see Chavira et al. 2016).

Academic goals: Adolescents’ academic goals were measured using their reports on academic goals from the semi-structured interviews.

### 2.5. Analytic Strategy

Grounded theory (Glaser and Strauss 1967; Corbin and Strauss 2015) and thematic analysis were conducted to analyze pre-program and post-program data to generate emerging themes across time of measurement (pre, post) and reporter (parent, adolescent) based on patterns in parents’ and adolescents’ interviews. We built on the existing categories of parental involvement for the post-participation analyses. Through the themes that were emerging from the interviews, we recognized that some themes of parental involvement overlapped with Tara Yosso’s Community Cultural Wealth Model (Yosso 2005) whose themes of capital were previously derived from her qualitative analyses. Therefore, we also employed thematic analyses and explained the themes in the context of CCW forms of capital in parents’ and adolescents’ reports of parental involvement within the home and school context. Adolescent’s academic goals were assessed based on the goals they described in the interviews.

Based on a model from Charmaz (2006), iterative inductive and deductive processes of open coding were employed. Common categories were clearly defined with inclusive/exclusive boundaries, were elaborated with examples (Corbin and Strauss 2015), and synthesized with Yosso (2005) concepts of Community Cultural Wealth (CCW). Emerging categories of parents’ and adolescents’ reports of parental involvement were elaborated in the context of CCW forms of capital and represented overlap with Yosso’s CCW model.

To assess the main question of interest, the themes of parental involvement in parents’ and adolescents’ pre- and post-semi-structured interviews and their relation to child’s reports on academic goals were examined. The pre- and-post-program data analysis allowed us to examine how (1) parents’ reports on parental involvement were associated with adolescents’ reports on academic goals; (2) how adolescents’ reports on parental involvement were associated with adolescents’ reports on academic goals; and (3) how parents’ and adolescents’ parental involvement and adolescents’ academic goals may have changed after participating in the program. This led to a second phase of grounded theory, axial coding (Corbin and Strauss 2015) was employed to examine the association between parents’ and adolescents’ reports of parental involvement and adolescents’ academic goals. Specifically, the axial grids were used as a form of visualization to answer the research questions and better understand the association between parental involvement and adolescents’ academic goals. For the axial coding at pre-program, first parents’ reports of parental involvement were ranked from lowest to highest in reported parental involvement. Adolescents’ reports of parental involvement were also ranked from lowest to highest. The ranking of parental involvement was based on parents’ and adolescents’ reports on communication, home supervision, school contact, and participation. Parental involvement was categorized into high when parents’ and adolescents reported more of the different forms of parental involvement; medium parental involvement was categorized based on some of the reports of parental involvement; low parental involvement was categorized as reporting few experiences of parental involvement. Adolescents’ academic goals were categorized as low if adolescents reported high school/some college; medium if adolescents reported bachelors/masters; and high if adolescents reported professional school/doctoral degree. The first author and a trained graduate researcher ranked the categories of adolescents’ and parents’ reports of parental involvement separately for inter-rater reliability (Corbin and Strauss 2008; Armstrong et al. 1997). Third, after each researcher ranked each parents’ and adolescents’ statements of perceived parental involvement, they discussed how participants fit within the category of low, medium, or high based on the themes and properties of parental involvement that emerged in the parent interviews. Adolescents’ reports of parental involvement were also categorized based on the themes that emerged in adolescents’ interviews. Fourth, selective coding (Corbin and Strauss 2015) was conducted after the axial grids were developed. Selective coding is the process of building an explanation that connects the categories or themes that emerged in the interviews, quadrant by quadrant, that incorporates all of the participants’ narratives. The final step of building a grounded theory was left out and is not included as part of the analysis.

## 3. Results

Properties of parental involvement represented in the data included communication with teachers (e.g., attending parent–teacher meetings and back to school night), communication with adolescent about school and academic goals, seeking academic resources in the school, at home, or outside of school, and giving their child life advice. Parents’ and adolescents’ reports of parental involvement were coded and categorized into low, medium, and high. High parental involvement included parents who reported more of the different forms of parental involvement that included attendance to parent–teacher meetings, were involved in school activities, asked their child questions about school, sought resources for their child through tutoring services or educational support, and gave their child life advice. Medium parental involvement included parents who reported having some communication with teachers, some involvement in school, some communication with their adolescent about school, some involvement at home, and sometimes seeking resources at school or outside of school, and sometimes giving life advice to their child. Low parental involvement included parents who had very little to no communication with teachers, were not actively involved in their child’s school, rarely communicated with adolescents about school, were not very involved at home, did not state many experiences about seeking resources in school or outside of school, and did not mention giving life advice to their child.

**Figure 1.**
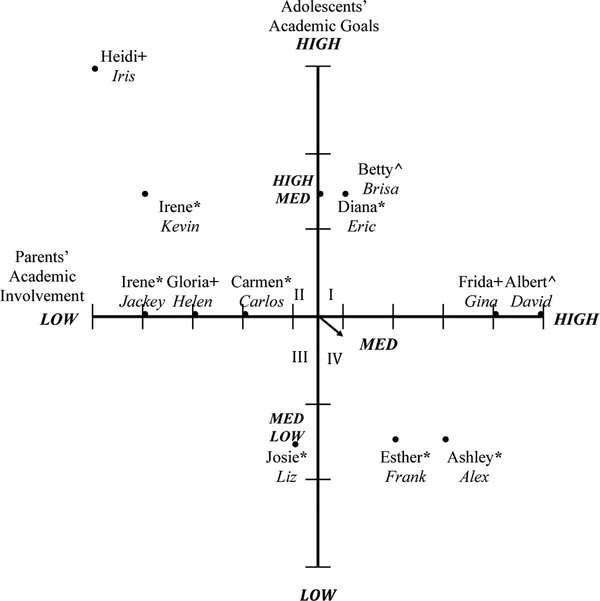
Parents’ academic involvement and adolescents’ academic goals pre-program. Adolescent pseudonyms are in italics. Parents education level: * = 6th grade or less; + = high school; ^ = graduated college/university. Quadrant I, four parents reported medium to high parental involvement and their adolescents reported medium to high academic goals. Quadrant II, five parents reported low to medium parental involvement and their adolescents reported medium to high academic goals. Quadrant III, one parent reported low to medium parental involvement and their adolescent reported low to medium academic goals. Quadrant IV, two parents reported medium to high parental involvement and their adolescent reported low to medium academic goals.

**Figure 2.**
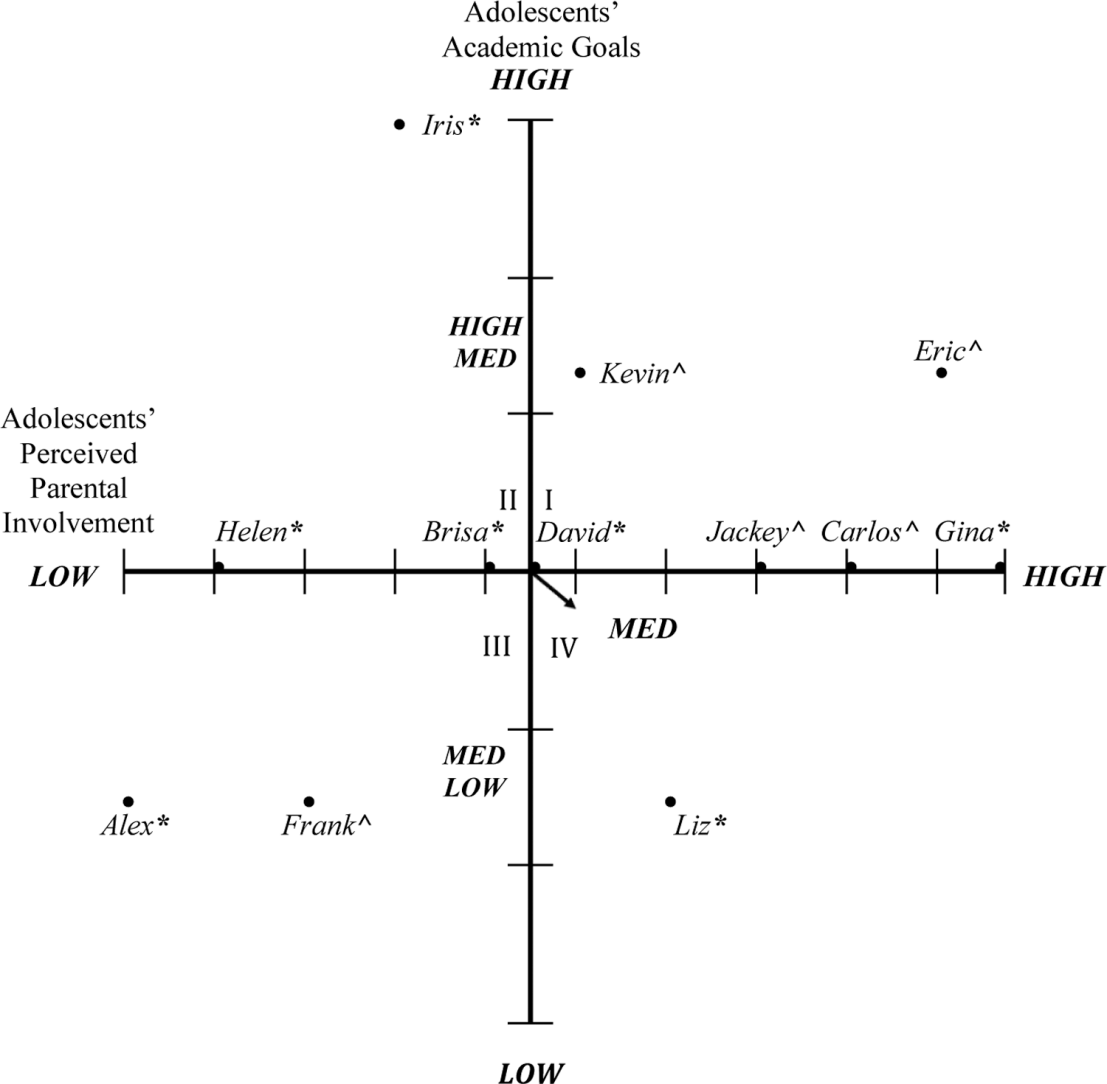
Adolescents’ perceived parental involvement and academic goals pre-program. Adolescent’s grade level: * = middle school; ^ = high school. Quadrant I, six adolescents reported medium to high parental involvement and reported medium to high academic goals. Quadrant II, three adolescents reported low to medium parental involvement and reported medium to high academic goals. Quadrant III, two adolescents reported low to medium parental involvement and reported low to medium academic goals. Quadrant IV, one adolescent reported medium to high parental involvement and reported low to medium academic goals.

**Figure 3.**
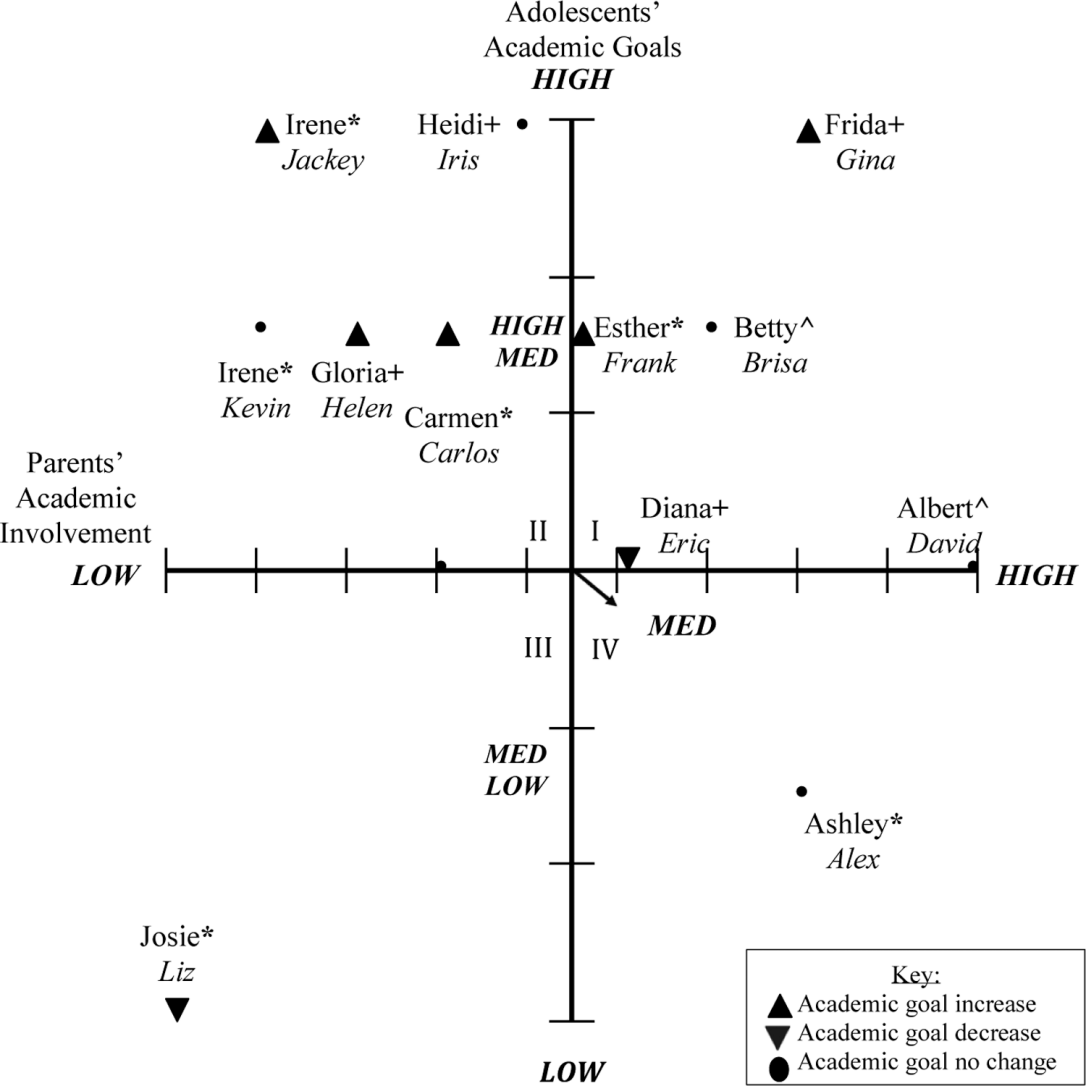
Parents’ academic involvement and adolescents’ academic goals post-program. Parents education level: * = 6th grade or less; + = high school; ^ = graduated college/university. Quadrant I, five parents reported medium to high parental involvement and their adolescents reported medium to high academic goals. Quadrant II, five parents reported low to medium parental involvement and their adolescents reported medium to high academic goals. Quadrant III, one parent reported low to medium parental involvement and their adolescent reported low to medium academic goals. Quadrant IV, one parent reported medium to high parental involvement and their adolescent reported low to medium academic goals.

**Figure 4.**
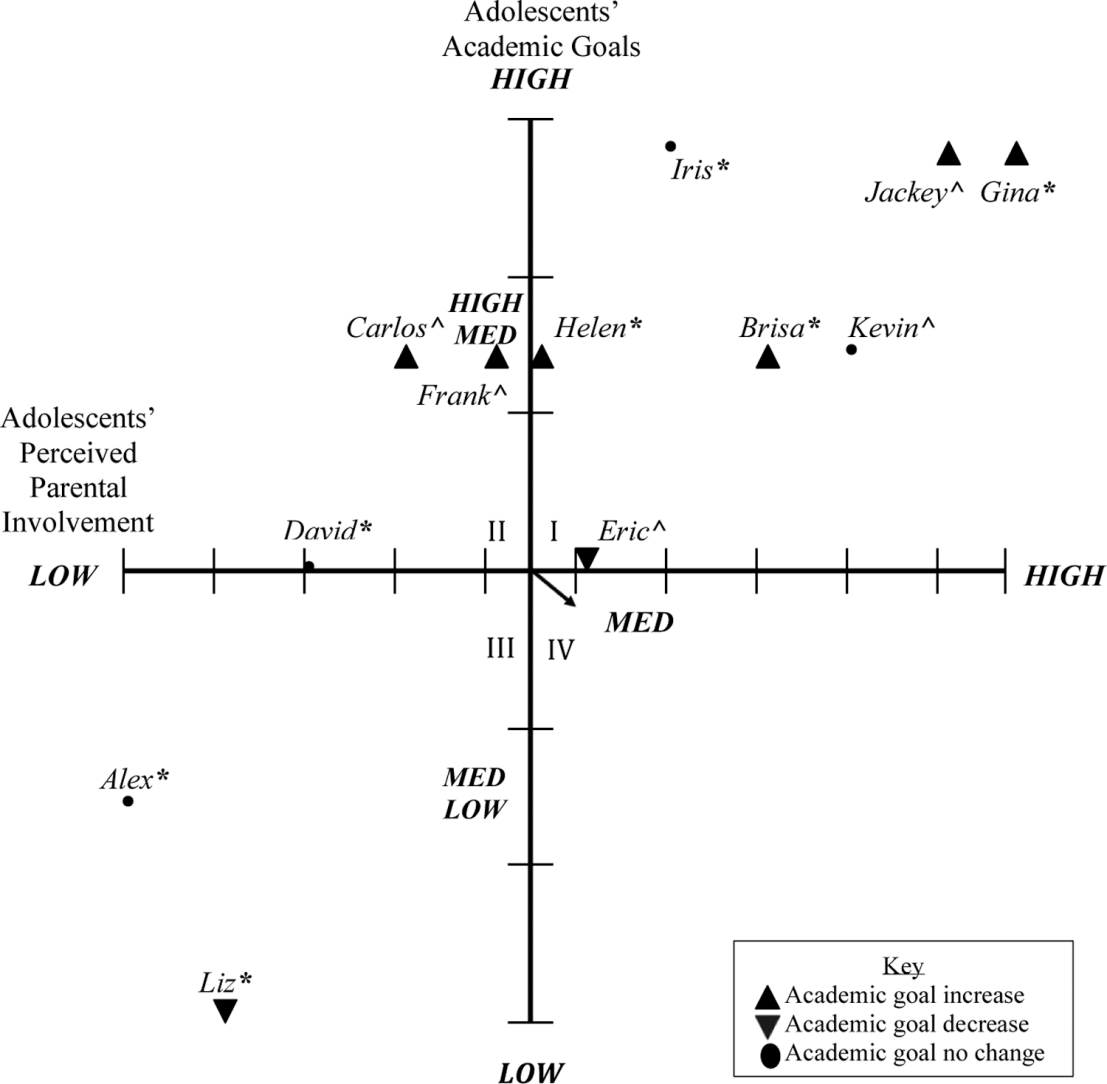
Adolescents’ perceived parental involvement and academic goals post-program. Adolescent’s grade level: * = middle school; ^ = high school. Quadrant I, seven adolescents reported medium to high parental involvement and reported medium to high academic goals. Quadrant II, three adolescents reported low to medium parental involvement and reported medium to high academic goals. Quadrant III, two adolescents reported low to medium parental involvement and reported low to medium academic goals. Quadrant IV, none of the adolescents reported medium to high parental involvement or low to medium academic goals.

Adolescents’ academic goals from the pre- and post-program interviews were coded and characterized into low (high school), medium-low (college), medium (university) medium-high (masters), and high (professional school/doctoral studies). For perceived parental involvement, adolescent interview responses were also analyzed and ranked from low to high parental involvement. Parents’ perceptions of their involvement and adolescents’ academic goals were included in Figure 1 (pre-program). Adolescents’ perceptions of parental involvement and adolescents’ academic goals were included in Figure 2 (pre-program). Parents’ and adolescents’ post-program perceptions of parental involvement and adolescents’ academic goals were presented in Figures 3 and 4, respectively. Parents’ and adolescents combined pre- and post-program perceptions of parental involvement and adolescents’ academic goals are also described.

### 3.1. Parents’ Perceptions of Parental Involvement and Adolescents’ Academic Goals (Pre-Program)

The first axial grid represented the selective coding of the association between parents’ academic involvement and their child’s academic goals before participating in the college knowledge program, see Figure 3. Quadrant I represented four parents who demonstrated medium to high parental involvement and their adolescents who reported medium (university) to medium-high (masters) academic goals. These parents reported consistently attending parent–teacher meetings, were involved in school activities, frequently asked their child questions about school and their academic goals, frequently sought resources for their child through tutoring services or educational support, and frequently gave their child life advice. To illustrate Quadrant I families, Albert, a college-educated parent, was highly involved in his special education son David’s middle school education, reported aspiring for David to attend a four-year university. He stated:

Well, what I have done with him is help him up to this point with all of his homework, to explain it to him so that studying becomes useful, going to school, reading. Obviously, all of that helps him to accomplish his goals. To study, to have a life that as a student, he always has to study. That’s what I’ve done.

When asked about his school involvement, Albert responded:

I am involved with his homework. In getting to know his teachers, talking with them. With any homework that perhaps, I pass it to him. Well first of all the assistant is also there at school. I’ve asked her to please help me with each one of his studies, that she makes note of his homework or activities, or anything that is pending. Because I cannot be in class all the time. There are days that I go with him since the morning, I listen to the class, I am with him, I see how he acts, and the assistant is there. We go to the next class and I am there with him all day. So in that sense, I get all the information daily and I always help with his homework.

According to Yosso (2005), Alberto demonstrated social capital by creating ties and relationships with school personnel to help his child do well in school. He also holds aspirational capital by maintaining hope for his child to succeed in school and navigational capital by identifying methods that will grant him the opportunity to obtain assistance for his child.

Quadrant II represented five parents who demonstrated medium to low parental involvement and adolescents who reported medium (university), medium-low (masters), and high (medical school) academic goals. These parents indicated having some or no communication with teachers, some or no involvement in school or communication with their adolescent about school, some or no involvement at home and sometimes seeking for some resources at school or outside of school, and little to no life advice. Quadrant II families are illustrated by Irene, a parent with an elementary education or less, demonstrated low academic parental involvement in her high school son Kevin’s education; Kevin reported aspiring to attain a master’s degree. She stated:

Well, we only give them advice as parents, and that they focus on their studies that they’re not going on to do other bad things; that is having problems at school or something like that. And until now, thank God, they have been good. We haven’t had complaints about something like that.

When asked about her involvement at Kevin’s school, Irene responded:

Ah, truthfully no, because I am not going to the school. I do go to all the [pause] when there is this, how do you say it? What is it when there are things for parents or so, but grand events, when they have back to school night, back to school.

Irene represents a sense of aspirational capital by giving advice to her children and communicating with them the value that she places on attending school by telling her children to focus on their studies yet is not involved at her son’s school or can articulate clearly her form of involvement.

Quadrant III represented one parent who demonstrated low-medium parental involvement and an adolescent who reported medium-low (community college) academic goals. This would include parents who had some communication with teachers, some involvement in school and at home, some communication with their adolescent about school, sought some academic resources, and demonstrated some life advice. Quadrant III is illustrated by Josie, a mom with an elementary education level or less, and who demonstrated medium-low parental involvement and whose middle school daughter Liz reported aspiring to attend a community college. She stated:

Sometimes they have tutoring. I tried to sign the paper so that at school they get tutoring and sometimes, sometimes, I would go walking [to school]. I used to have a ride and she would stay after school for tutoring. And before, I used to go walking to pick her up as long as she stayed in tutoring and well. I also try to, like now here at [CBO], I have tutoring for them.

When asked about her involvement in Liz’s school, Josie has found it challenging to be involved in both of her children’s schools. She responded:

In her school, not much [involved] because it is sometimes difficult for me to go, but the school I am very involved in is the one that is here on (street name), that one, yes. Sometimes at the other school, it is difficult for me because it is further away and I have to come to pick up my other children and make them food and like that right, but this is the one that is closest to me.

According to Yosso (2005) CCW, Josie holds navigational capital by utilizing the tools and resources in her surrounding such as signing forms for tutoring services and seeking tutoring services outside of school to help her child do well academically. Josie identified methods to indirectly assist Liz in her school work through services available in her child’s school and her community. Josie also highlights some of the challenges she experiences in terms of transportation to Liz’s school, which she states has made it difficult to go to her school. This could explain why she may not be highly involved in Liz’s school. It could also be that parents with children at different grade levels find it challenging to be involved in multiple schools and often choose one, as Josie has done.

Quadrant IV represented two parents who demonstrated medium to high parental academic involvement and adolescents who reported medium-low (community college) academic goals. These parents had some communication with teachers, some involvement in school and at home, some communication with the adolescent about school, sought some academic resources at school or outside of school and demonstrated some life advice. Quadrant IV families are illustrated by Ashley, a parent with an elementary education or less and whose middle school son Alex reported aspiring to attend a community college, demonstrated medium-high academic parental involvement. She stated:

My other family, well no [other family members do not help Alex reach his goals]. But I always bring him [to CBO] and my husband also, well I think that he supports me, because well he says, “any decision that you make for the boy, while you think that it is good for the boy then I will support you”.

When asked about her school involvement, Ashley responded:

When there are conferences like I told you they don’t call me, I am present anyway. When there’s something like that where they are going to give information, I always go. If I can, I always go. When there are workshops for parents because I have my baby, I can’t. But yes, I try to force myself when there is something where I have to be there present.

According to Yosso (2005), Ashley demonstrated navigational capital by employing a sense of agency and demonstrating an awareness of school-related meetings and attending these events. This parent also demonstrated some challenges she experiences, such as having a baby or not always being informed of school events, which may be related to the medium parent academic involvement that was demonstrated. The mother’s responsibilities could have also resulted in circumstances that may not always allow her to be present and involved in Alex’s school.

These findings indicated that parental academic involvement prior to their participation in the college knowledge program ranged from low to high such as having high involvement in their child’s academic experiences at home and a school (high), having some involvement in their child’s academics (medium), or having little involvement in their child’s education at home and at school (low). Adolescents’ goals ranged from attending community college (medium-low) to pursuing professional school/doctoral studies (high) before participating in the program.

### 3.2. Adolescents’ Perceptions of Parental Involvement and Adolescents’ Academic Goals (Pre-Program)

To examine the associations between the adolescents’ perceived parental involvement and adolescents’ academic goals before participating in the college knowledge program, the adolescents’ perceived parental involvement was defined through their responses to their parents’ home and school involvement. Properties of perceived parental involvement included communication with teachers (e.g., attending parent–teacher meetings and back to school night), communication with their adolescent about academic goals, life advice, and seeking academic resources in the school, at home, or outside of school. Adolescents’ reports of perceived parental support were characterized into low, medium, and high. High parental involvement included more of the different forms of parental involvement such as adolescents’ statements of parents who consistently attended parent–teacher meetings, were involved in school activities, asked them about school, sought resources for them through tutoring services or educational support at school or outside of school, and gave them life advice. Medium parental involvement was included adolescents’ reports on parents who were perceived as having had some communication with their teachers, some involvement in school, some communication with them about school, some school-related involvement at home, and sometimes seeking resources for them at school or outside of school, and sometimes giving them life advice. Low parental involvement was included reports of parents who were perceived as having very little to no communication with their teachers, were not actively involved in their school, did not communicate with them about school, were not academically involved with them at home, did not state any experiences about parents seeking resources for them in school or outside of school, and did not state any experiences with their parents giving them life advice. Adolescents’ academic goals were also characterized into low (high school), medium-low (college), medium (university), medium-high (masters), and high (professional school/doctoral studies).

An axial grid was developed to represent the associations between adolescents’ perceived parental involvement and their academic goals before participating in the college knowledge program, see Figure 2. Quadrant I represented six adolescents who perceived medium to high parental involvement and reported medium (university) to high (masters) high academic goals. These adolescents stated that their parents attended parent–teacher meetings, were involved in school activities, asked them questions about school and their academic goals, sought resources for them through tutoring or educational support, and gave them life advice. To illustrate, Gina, a middle school student who aspired to attend a university, reported high levels of perceived parental involvement when asked what her family is doing to help her achieve her goals:

Yes, my family has been helping me achieve my goals, like whenever I need help from the library, they make that effort to take me there. Or like when I need help on something, they find a way to tutor me. They are like helping me with everything that I am having trouble with so that I can excel in it.

When asked how her parents are involved in school, Gina stated:

My dad not really, but my mom (Frida) is involved; she volunteers. She already has her badge and she volunteers in field trips, school and meeting, she is part of meetings.

According to Yosso (2005), this adolescent demonstrates the social capital available to her by her parents’ actions to seek educational services that will help her do well in school. Furthermore, the adolescent highlights the aspirational capital in her family by stating that the purpose behind her parents’ involvement is for her to excel in school. This also presents the navigational capital endorsed in her family by navigating school assignments through the assistance of resources being sought by her parents.

Quadrant II demonstrates three adolescents who indicated medium to low parental involvement and adolescents who stated medium (university) to high (medical school) academic goals. These parents indicated having some or no communication with teachers, some or no involvement in school, some communication with their adolescent about school, some involvement at home, seeking some resources at school or outside of school, and some-to-no life advice. Helen, a middle school student who aspired to attend a university, reported low perceived academic parental involvement when asked how her parents are helping her achieve her goals:

Well right now my dad is, well, since I am working really hard and getting good grades my dad is like letting me relax during the weekend but my mom is, like, she is not always going to be there for me cause you know after when you get older and stuff, that I am going to have to do my things by myself, so she is making me do chores and I am trying to learn to cook.

When asked about how her parents are involved at school, Helen stated:

No [no they are not involved] … Well, my mom would. In elementary, my mom would help out with activities and stuff.

This adolescent was ranked as having low perceived parental involvement. However, despite showing low perceived parental involvement compared to other adolescents, this adolescent highlights the familial capital present in her family through her parents’ messages about learning responsibilities early on for her well-being in the future.

Quadrant III demonstrates two adolescents who stated low to medium parental involvement and medium-low (community college) academic goals. These parents demonstrated some or no communication with teachers, some or no involvement in school, some communication with their adolescent about school, some involvement at home, seeking for some resources at school or outside of school, and some to no life advice. Frank, a high school student who aspired to attend a community college, demonstrated low-medium perceived parental involvement when asked what his parents are doing to help in achieving his goals by stating: “Telling me to keep my head in school, um and telling me to, (pause) I guess tutor here [at CBO].” When asked about how his parents are involved in school, Frank stated: “Yeah. They go check my grades and stuff like that. Um, when there are conferences. Um, it usually depends when, like out of random.”

According to Yosso (2005), Frank demonstrates a sense of aspirational capital as afforded through his parents’ statements of wanting him to stay focused in school. However, compared to other adolescents’ reports of parental involvement, he demonstrated medium-low perceived involvement from his parents.

Quadrant IV includes one adolescent who indicated medium to high parental involvement and medium-low (community college) academic goals. This would include parents who had some communication with teachers, some involvement in school and at home, some communication with their adolescent about school, sought some academic resources, and demonstrated some life advice. Liz, a middle school student who reported aspiring to attend a community college, demonstrated low-medium levels of perceived academic parental involvement, when asked how her parents were helping her achieve her goals. She stated:

Yeah, they told me that you have to go to school, get good grades to be like someone not to be like other people that don’t really like school.

When asked about how her parents are involved in school, Liz stated:

Just my mom (Josie), like she helps the school.

According to Yosso (2005), this adolescent demonstrates a sense of aspirational capital through the messages that this adolescent reported receiving from her parents to go to school and do well in school.

These findings revealed that before participating in the college knowledge program, nine adolescents who reported low to high levels of parental involvement indicated having medium (university) to high (professional school/doctoral studies) academic goals. Three adolescents, who reported low to high levels of parental involvement, indicated medium-low (community college) academic goals before participating in the program.

### 3.3. Parents’ Perceptions of Parental Involvement and Adolescents’ Academic Goals (Post-Program)

A third axial grid represented the association between parents’ academic involvement and their children’s academic goals post-college knowledge program, see Figure 3. In Quadrant I, five parents were represented and these were the same parents represented in Quadrant I at pre-program. In this quadrant, adolescents reported medium (university), medium-high (masters), and high (doctoral studies/medical school). After these parents participated in the program, they continued to attend parent–teacher meetings, were involved in school activities, continued to ask their child questions about school and academic goals, and sought resources for their child through tutoring or educational support, and gave life advice to their child. To illustrate a parent who remained in Quadrant I, and whose daughter attended middle school and went from aspiring to attend a university (T1) to aspiring to pursue a doctoral degree (T2) when asked what she or her family does to help Iris achieve her goals, Frida stated the following:

My husband is a little bit more [pause] he needs a little more guidance on how important it is to be on time. He is like no [pause] and I explain to him, you do not know how important it is for the children to arrive on time, apart from that I learned that a child when you are late goes anxiously to school, goes nervous, and goes with stress so early in the morning. So, I talked to my husband and I told him, ‘you know that if Gina is going to go to a school that is further away we will organize, I will take the little one’, because they go to different schools … Because, in fact, that is where discipline and responsibility begin as parents. Then I tell my husband, ‘if you are going to commit to take her on time, get up because it is late and get up because it is late.’ Because he has a hard time getting up … So that’s what we’re doing at home, taking our daughter on time. And when she has to do projects I say, ‘[father name] the girl is going to do a project, we need to buy this and that.’ And sometimes he takes us … We as parents, as a family, providing at home what the child, well, what the girl needs for her study.

When asked about her school involvement, Frida responded:

Definitely [involved in school]. Definitely because now, I already, my daughter is now in middle school so I have to be aware that not because she is growing up she is becoming lazy at school [chuckles] that is what I tell her, daughter you’re growing, you’re bigger, you understand more now how important it is to maintain your good grades. Because do not say, ‘oh I’m going to be lazy.’ I tell her the word lazy, ‘I’m going to be lazy after all I still have a lot of time left.’ And that’s the problem, there’s the dilemma. And what? That there [at school] they start to fall behind and fall behind, and to straighten up, it is going to cost [her] … As a mother, as parents, we are, we hope that our children listen to us. I tell my daughter, so that life does not complicate so much. Because it cannot be so complicated, it can be more bearable, a simpler life, if not complicated from the beginning.

In Quadrant II, five parents were represented in the medium-low parental involvement and adolescents reported medium medium-high (masters) to high (doctoral studies/professional school). These parents indicated having some or no communication with teachers, some to no involvement in school and at home, some to no communication with their adolescent about school, sought some academic resources, and demonstrated little to no life advice. To illustrate, Heidi, a parent whose daughter Iris attended middle school, and indicated aspiring to pursue medical school, went from demonstrating low parental involvement (T1) to medium academic parental involvement (T2). When asked what she or her family does to help Iris achieve her goals, she stated:

Well, I take her more often to the library so she takes more of the habit of reading and I’m motivating her to introduce herself to a sport thing that they can help her meet the requirements and I’ve shared with my husband I say, ‘It’s not about just saying I want you to be what I want, no.’ As parents, we also have to put our part not just say it and be there. ‘Daughter I am here when you need me, where do you have to go to get your credits? Whatever it is, I’m going to take you or if not just telling them I want you to get there and put your all in it’, it’s not enough. That is what I shared with my husband. I say I did not know many things, but thank God I’m learning, and there is still time to learn more I tell him.

When asked about her school involvement, Heidi responded:

Yes, I always avoid forgetting to ask her, ‘What did you do today?’ and sometimes when I pick her up when she gets in the car, in the morning she says that she is going to have an exam and I ask her how did the exam go? And sometimes she takes the initiative to tell me, ‘oh you know the exam was very easy and but it is because I study’. She tells me so. I say, ‘You see.’ I think I have seen that difference in her that she is studying because sometimes she would be satisfied with that, ‘I already know.’ She did not review and now [with] the last exam she tells me the exam was very easy. I believe that maybe what she learned has motivated her.

In Quadrant III, one parent was represented who reported low parental involvement and whose adolescent reported low academic goals (high school). Quadrant III included parents who had some to no communication with teachers, some to no involvement in school and at home, some to no communication with their adolescent about school, sought some or no academic resources in school or outside of school and demonstrated little to no life advice. To illustrate the parent in Quadrant III, Josie, a parent who reported a 6th-grade education level or less and whose daughter attended middle school and aspired for a high school diploma, described what she and her family did to help her academically, “Well, how, well yes, well, take them to class and bring them to tutoring, then yes, yes we are [helping Liz achieve her goals]. Well, yes I think so (laughs).” When asked about her school involvement, Josie responded, “Not in her school, but the school of the other children, yes. In her school, no [not involved] because it is far for me.”

Quadrant IV represented one parent who reported medium-high parental involvement after participating in the program and the adolescent reported low-medium (community college) academic goals. This quadrant included parents who had some communication with teachers, some involvement in school and at home, some communication with their adolescent about school, sought some academic resources, and demonstrated little to no life advice. To illustrate this quadrant, a mother, Ashley who reported a 6th-grade education level or less, represented medium-high parental involvement. Her son, Alex attended middle school and aspired to attend a community college. Below, she described what she and her family did to help Alex academically:

Checking how he is doing at school, his grades every eight days, seeing how he is doing. Like, if he turns in his homework. If he did homework. Bringing him here [CBO] for tutoring for them to help him. And go to conferences with teachers.

When asked about her school involvement, Ashley responded:

Yes, I have looked for more help. More help in asking for information with the teachers. [pause] Yes, I am always aware of him. Yes … ‘Do you need help,’ even here at tutoring I talk with the tutor or with (coordinator), ‘You know Alex needs more here, I need them to help him here [subject],’ or I go with the teachers. ‘Why did this happen?’ And ‘What can we do to help him.’

### 3.4. Adolescents’ Perceptions of Parental Involvement and Adolescents’ Academic Goals (Post-Program)

A fourth axial grid was created to represent the association between adolescents’ perceived parental involvement and their own academic goals after the college knowledge program, see Figure 4. Quadrant I represented seven adolescents who reported medium to high parental involvement. These adolescents stated that their parents attended parent–teacher meetings, were involved in school activities, asked them questions about school, academic goals, sought resources for them through tutoring or educational support, and gave them life advice. Adolescents in this quadrant also reported medium (university) to high (doctoral studies/professional school) academic goals. To illustrate, when asked what her family is doing to help her achieve her goals, Gina, a middle school student who reported aspiring to attend a university pre-progam and reported aspiring to pursue doctoral studies during the post-program interview, demonstrated high levels of perceived academic parental involvement by stating:

Yes, like sometimes we get tutoring or, sometimes um they help me with homework. So, my mom is taking high school, like she’s finished high school but she’s still taking some classes. She helps me or sometimes I can help her. So, we’re like, so we’re like working together to like, achieve our goals.

When asked how her parents are involved in school, Gina stated the following:

Uh, they’ve pretty much stayed the same and they’re pretty close, so yeah. Um, they’re involved, my mom. She’s usually attending district meetings or she’s checking out ‘oh this high school is pretty good’ or sometimes I could be like ‘oh hey mom there’s this program in school. Can I join it.’ And my dad, he usually provides us, ‘okay, here’s some money’ and likes to attend the program.

Quadrant II demonstrates three adolescents who reported medium to low parental involvement and medium (university) to high (masters) academic goals. Adolescents also reported medium-low (college) to medium-high (university) academic goals. These adolescents perceived their parents as having some or no communication with teachers, some or no involvement in school, some communication with their adolescent about school, some involvement at home, sought for some resources at school or outside of school, and gave them some life advice. To illustrate, when asked what his family is doing to help him achieve his goals, Frank, a high school student who reported aspiring to attend a community college education before beginning the program and aspiring to pursue a master’s degree after participating in the program, demonstrated medium levels of perceived academic parental involvement:

Um, most likely my mom … she, yeah, she has changed. She thinks twice about the things she says [be]cause you know it’s not that easy. Like sometimes they would leave me homework and sometimes they won’t so when, when they do and they see me work all night. They know I’m doing something you know.

When asked about how his parents were involved in school, Frank stated:

They’ve been involved before, yeah, in school. They were asked since my mom was, used to work for the school. Yes, so she, she basically knows everyone there so honestly, she is not going to know (answered laughing). Yes, of course. Yeah, asking for my grades, yeah.

Quadrant III represented two adolescents who reported medium-low perceived parental involvement. Adolescents also reported medium-low (community college) and low (high school) academic goals. These adolescents perceived their parents as having some or no communication with their teachers, some or no involvement in school, some or no communication with them about school, some involvement at home, sought for some resources for them at school or outside of school, and gave them some life advice. To illustrate an adolescent in Quadrant III, Alex a middle school student reported low parental involvement and medium-low (community college) academic goals. When asked what his family is doing to help him achieve his goals, Alex stated: “They’re helping. They take me to tutoring.” When asked how his parents are involved in school, Alex stated the following: “They called the teachers to see how I was doing, yeah.”

Quadrant IV represented medium to high parental involvement and medium (university) to low (high school) academic goals. No adolescents were represented in quadrant IV. Overall, 10 of the 12 adolescents who were in the first two quadrants indicated low to high levels of perceived parental involvement and medium (university) to high (doctoral studies/medical school) academic goals after participating in the college knowledge program.

### 3.5. Changes in Parents’ Perceptions of Parental Involvement and Adolescents’ Academic Goals

Changes in parents’ reports of parental involvement and adolescents’ reports of academic goals emerged from pre-program to post-program. In Quadrant I, five parents remained in the medium to high level of parental involvement, however, increases in adolescents’ academic goals emerged from community college to university and from university to doctoral studies. One family with a medium-high parental involvement had a slight decrease in adolescent’s academic goals from master’s to four-year university. A second family with high parental involvement had an adolescent who remained the same in their academic goals (four-year university). In Quadrant II, one parent increased in parental involvement from low to medium and the adolescents’ academic goals remained the same (medical school). There were also increases in adolescents’ academic goals from university to masters or university to doctoral studies/medical school.

Quadrant III represented one parent who decreased in parental involvement from medium to low and the adolescent decreased in her academic goals from medium-low (community college) to low (high school). Lastly, Quadrant IV demonstrates one parent who remained at the same level of parental involvement (medium-high) after participating in the program and the adolescent’s academic goal remained the same (community college).

The study’s findings demonstrated pre-post changes in parental involvement and adolescents’ academic goals. Overall, findings indicated that a majority of parental academic involvement remained relatively the same in the axial grid after participating in the college knowledge program. It is important to highlight that these families were involved in their adolescents’ academic experiences before beginning the college knowledge program and continued to be involved after participating in the program. Therefore, a shift in parental involvement for all parents may not be visible if parents continued their involvement. It is also important to highlight that parents added to their parental academic involvement.

### 3.6. Changes in Adolescents’ Perceptions of Parental Involvement and Adolescents’ Academic Goals

Changes in adolescents’ reports of parental involvement and their own academic goals emerged after participating in the program. For instance, in Quadrant I two adolescents (i.e., Carlos, David) shifted from medium-high to medium-low perceived parental involvement, however, the academic goals for one adolescent increased from medium (university) to high (masters) and for the other adolescent there were no changes in their academic goals. In Quadrant II, three adolescents (i.e., Helen, Iris, Brisa) shifted from medium-low perceived parental involvement to medium-high parental involvement, and for two of these adolescents, their academic goals also increased from medium (university) to high (masters), and for the other adolescent there were no changes in their academic goals. In Quadrant III, no adolescents shifted in their perceived parental involvement, however, one adolescent (Frank) increased in his academic goals from medium-low (community college) to medium-high (masters). Lastly, in Quadrant IV, one adolescent decreased in her level of perceived parental involvement from medium-high to medium-low and decreased in her academic goals from medium-low (community college) to low (high school).

## 4. Discussion

The current study examined how parents’ and adolescents’ reports of parental involvement were associated with adolescents’ academic goals before and after participating in a college knowledge program. After participating in the program, parents’ reports of their involvement suggested that their involvement remained relatively the same. Half of the adolescents indicated increases in their academic goals (university to master’s or professional school/doctoral studies) and went from reporting medium (pre-program) to high levels (post-program) of perceived parental involvement. This study’s findings indicated that after parents and adolescents participated in the program, shifts in perceived parental involvement and adolescents’ academic goals emerged. The current study findings also identify the possibility of a program to help families access additional capital that will allow them and their children to traverse through academic pathways and higher education.

It is important to highlight that all of the parents were involved in their child’s education before beginning their participation in the college knowledge program. After analyzing parents’ reports of their involvement at home and school before and after participating in the college knowledge program, 8 out of the 11 parents who participated in this study remained in the same quadrant for parental academic involvement. Specifically, two parents’ academic involvement increased after participating in the program. These parents reported a high school education level and a college education level. Parents’ education level may have been associated with their level of involvement and knowledge about the U.S. education system. The average education level for parents in this sample was 7.33 years. Therefore, it is also possible that these parents’ human capital may be associated with their familiarity with the process associated with pursuing higher education and how their involvement can continue to help their children. In contrast, two parents decreased in their involvement after participating in the program. These two parents had a 6th-grade education level or less, which was slightly below the average parent. It may be that these parents’ low levels of education may have played a role in how they continued their involvement in their child’s academic experiences. For example, not being familiar with U.S. formal schooling may limit their understanding of the processes of their involvement in their child’s education.

Another important factor to consider is parents’ attendance in the sessions. Parents’ attendance during the college knowledge program varied. The average attendance was 4.0 sessions for adolescents and 4.3 sessions for parents. More specifically, the parent who demonstrated a decrease in her parental involvement after participating in the college knowledge program (Josie) only attended two of the six sessions. It is possible that parents’ attendance was also associated with the information that parents learned and how they continued to employ their involvement in their child’s academic experiences. It is important to highlight that although a majority of parental academic involvement remained relatively the same after participating in the college knowledge program these families were involved in their adolescents’ academics before beginning the program and continued to be involved after participating. Furthermore, since these parents were already involved in their children’s academic experiences, after participating in the college knowledge session they continued or added to their existing parental involvement. Therefore, parents in the medium-to-high quadrant remained in the same quadrant after participating in the program. This finding parallels those of the Parent Institute for Quality Education on parent involvement where parents reported changes in their communication with their children and their teachers (Chrispeels and Rivero 2001). Parents who participated in PIQE also reported that they developed awareness on how to work with their children in related school activities and as a result, this led to an increase in their communication with their child. Therefore, the majority of the current families who participated in this study who were involved before attending the program continued their involvement, but they also enhanced or did more of what they were already doing such as communicating more with teachers or asking teachers or tutors more about their child’s academic progress.

Parents’ and adolescents’ reports of parental involvement also indicated the frequent forms of capital that existed within these families. Parents demonstrated aspirational capital, navigational capital, and social capital. This is important to highlight because aspirational capital demonstrates the hopes and dreams that parents have for their children to succeed despite challenges they may experience. Through this sense of aspirational capital families also demonstrated navigational capital as indicated by parents reports of how they do what they can to attend parent–teacher meetings, communicate with teachers about their child’s progress, and inquire about tutoring services. Through families’ reports of how they navigate with their children in school, these families also demonstrate a sense of social capital as parents report how they develop relationships with the school and tutoring centers. The majority of the parents held low levels of human capital, yet they held additional sources of capital as highlighted by the Community Cultural Wealth model (Yosso 2005) (i.e., goals, navigation, and social) that granted them the opportunity to remain involved in their child’s academic experiences. This is important to acknowledge when aiming to understand how parents remain involved despite not having first-hand experience with formal schooling in the United States.

These findings also highlight some changes in adolescents’ academic goals. For instance, one high school adolescent’s academic goals increased from medium-low (community college) to medium (university). Two adolescents increased from medium (university) to high (doctoral studies) and three adolescents increased from medium (university) to medium-high (masters) in their academic goals. These findings indicated that 6 of the 12 adolescents had an increase in their academic goals after participating in the program. Of these six adolescents with increases in academic goals, three were in high school and three were in middle school. It appears that there are no grade-level differences in academic goals, yet a larger sample size that includes both middle and high school students is needed to test for grade related differences in academic goals after participating in the program. One potential explanation for the changes that emerged in adolescents’ academic goals is that parents’ participation in these sessions may have changed their behaviors or awareness of college, that played a role in how they interacted with their children about school, which in turn could have served as a motivator for adolescents increased academic goals.

The findings on the adolescents’ perceived academic parental involvement revealed that 7 of the 12 adolescents reported medium to high parental involvement and medium to high academic goals after participating in the program. Four of these adolescents were in middle school and three were in high school. Although we are not able to test for grade-level differences between middle school and high school adolescents on their perceived parental involvement, these results demonstrate that high levels of perceived parental involvement exist for middle school and high school students. These results align with The Educational Resources Institute (TERI) report finding that college preparator information and guidance are major components in realizing students’ college goals (Vargas 2004). This 2004 report suggests that “interventions focused on providing information and guidance about college to underrepresented students and families, both early and often”, can provide them with much needed college knowledge. Therefore, developing a college knowledge program that targets all adolescents may be beneficial to students as early as middle school and not just high school, when most pre-college programs exist. These findings also suggest that adolescents’ perceived parental academic involvement is associated with adolescents’ academic goals for their future. That is, adolescents who perceived medium to high levels of parent involvement also reported medium to high levels of academic goals, which highlights a positive association between parental involvement and academic goals. It is unclear if parental involvement leads to higher academic goals for adolescents, however, previous studies have also found the same association (see Chavira et al. 2016; Carranza et al. 2009; Hill et al. 2004).

After adolescents participated in the program, six adolescents demonstrated an increase in their parents’ perceived involvement, while four adolescents decreased in their perceived parental involvement. It may be that for the four who had decreased parental involvement, they did not believe that their parents were as involved in their education (as they initially reported) after learning about all the steps and procedures needed to prepare for college admission. These adolescents may have realized that although their parents were involved at home and school, they were not providing them with the college knowledge due to their parents’ unfamiliarity with the U.S. educational system. A previous study focused on parents and adolescents in an engagement program found that the program served as a vehicle for parents’ development of their college knowledge and insight into their child’s academic experiences (Auerbach 2004). College knowledge programs are important for adolescents to gain an understanding of college admission procedures and without these programs, the information that adolescents receive could be less consistent (Auerbach 2004). Presenting parents and adolescents with the same information could have also served as a vehicle for these families to work together and communicate more about university admission requirements and resources available.

Previous studies highlight the value that Latino parents place on their children’s academic experiences and the pursuit of higher education (Lopez 2009). Due to the language and educational barriers that Latino families experience, many parents are often unable to help their children with homework or guide them towards a college path (Stanton-Salazar 2001). Despite these challenges, parents reminded their children to take advantage of what school has to offer them to be able to obtain a life that does not entail poverty or struggles (Stanton-Salazar 2001). This study highlights parents’ continuous academic involvement and the social, cultural, navigational, and aspirational capital that they possess to help their children strive for higher education and as many of these parents stated, work towards a “better life”.

The study findings can inform other community-based organizations to adopt similar college knowledge and preparation programs for parents and adolescents to help them and their children as they prepare to attend a post-secondary institution. Furthermore, these findings can inform how middle schools and high schools can capitalize on their students’ college knowledge and possibly implement programs for their students and their families to help them increase their college knowledge and awareness of the steps associated with college admission. These programs can also employ a parent component that informs parents on how they can continue their involvement in their child’s academic experiences.

Universities or colleges should also consider developing partnerships with middle schools and high schools and invest in a college knowledge program to increase students’ college knowledge. These findings can also inform school counselors about the information that should be made accessible for both parents and adolescents to develop a partnership between families and schools to help students apply to universities. In addition, the findings of this study can inform policies on the impact of a college knowledge program and inform them of the value of providing funding for middle schools, high schools, and community-based organizations to deliver a college knowledge program for families.

## 5. Limitations and Future Studies

The current study findings indicated changes in parents’ and adolescents’ reports of parental involvement and changes in adolescents’ academic goals, but results must be considered in light of some limitations. For instance, a total of 11 parents and 12 adolescents participated in the current study and as a result, these findings cannot be generalizable to the larger Latino population. Furthermore, this study took place in the greater Los Angeles area and may not be representative of Latino populations in other geographical locations. This study included a majority of families who were participants of a community based non-profit organization that aims to provide families with educational training opportunities, thereby, indicating that these families were already involved in some form of academic involvement with their child. This may not be the case for all parents who do not have a community-based organization with similar services in their communities. Also, study findings indicated that adolescents in middle school and high school both increased in their perceived parental involvement and academic goals. Future studies should include a larger sample size to identify the significance of students’ grade level on their academic goals and perceived parental involvement.

Participants varied in their attendance ranging from two to six sessions, which was not examined in this study. Future research should consider parents’ and adolescents’ attendance in a college knowledge program to measure how attendance plays a role on college knowledge gains and changes in parent involvement and academic goals. Also, each program session lasted 1.5 h, every three weeks, for a total of nine hours for the whole program. Given that the program was extended across 18 weeks it is possible there could be maturational threats to validity. For example, adolescents could have been receiving college knowledge information from additional sources such as a mentor or counselor in the students’ lives, that were not accounted for; therefore, it is possible that students’ academic goals increased after the program because of extraneous variables that were not measured in the study.

Future studies should aim to examine the long-term impacts of a college knowledge program and investigate if parental academic involvement remained and persisted throughout the high school years. In addition, a longitudinal approach can help researchers and educators better understand the impact of this type of program on adolescents’ grades, completion of courses required by California universities (A-G requirements), and university enrollment. A follow-up study should also examine how much information families can retain in the future and how parents and students are applying the tools they learned.

## 6. Conclusions

The current study adds to existing knowledge of college knowledge programs for parents and their children. The goal of this study was to capture changes in parental involvement and academic goals before and after participating in a college knowledge program in a community-based organization for parent-adolescent dyads. Additionally, through parents’ and adolescents’ reports, we were able to better understand the sources of capital that families possessed, which further indicated the different levels of parental involvement that may not always be tangible and often go unnoticed. This study shines light on the possibility to help families’ access additional capital that will allow them, and their children traverse through the educational pipeline and together achieve the academic goals to which many of these families aspire.
